# Spectroscopic Characterization of the Product Ions Formed by Electron Ionization of Adamantane

**DOI:** 10.1002/cphc.201800846

**Published:** 2018-10-30

**Authors:** Jordy Bouwman, Stefan Horst, Jos Oomens

**Affiliations:** ^1^ Radboud University Institute for Molecules and Materials, FELIX Laboratory, Toernooiveld 7 NL-6525 ED Nijmegen the Netherlands; ^2^ Present address: Sackler Laboratory for Astrophysics, Leiden Observatory Leiden University P.O. Box 9513 2300 RA Leiden The Netherlands; ^3^ van't Hoff Institute for Molecular Sciences University of Amsterdam Science Park 904 1098XH Amsterdam The Netherlands

**Keywords:** carbocations, density functional theory, IR spectroscopy, mass spectrometry, structure elucidation

## Abstract

A structural characterization of the products formed in the dissociative electron ionization of adamantane (C_10_H_16_) is presented. Molecular structures of product ions are suggested based on multiple‐photon dissociation spectroscopy using the Free Electron Laser for Infrared eXperiments (FELIX) in combination with quantum‐chemical calculations. Product ions are individually isolated in an ion trap tandem mass spectrometer and their action IR spectra are recorded. Atomic hydrogen loss from adamantane yields the 1‐adamantyl isomer. The IR spectrum of the C_8_H_11_^+^ product ion is best reproduced by computed spectra of 2‐ and 4‐protonated *meta*‐xylene and *ortho*‐ and *para‐*protonated ethylbenzenes. The spectrum of the product ion at *m/z* 93 suggests that it is composed of a mixture of *ortho*‐protonated toluene, *para*‐protonated toluene and 1,2‐dihydrotropylium, while the spectrum of the *m/z* 79 ion is consistent with the benzenium ion. This study thus suggests that adamantane is efficiently converted into aromatic species and astrophysical implications for the interstellar medium are highlighted.

## 1 Introduction

Nanodiamonds, or diamondoids, are molecules that consist of *sp* ^3^ hybridized carbon atoms arranged in a diamond lattice structure terminated by hydrogen atoms. They comprise a relatively novel class of molecules with a variety of potential applications in biochemistry, pharmaceutical sciences and nanotechnology.^1^, ^2^ Their physical and chemical properties have been subject of a large number of recent investigations. Small nanodiamonds occur naturally and are found in hydrocarbon mixtures such as crude oil and natural gas and can be isolated from those resources,^3^, ^4^ but they can also be synthesized by a variety of methods.^5^, ^6^

Nanodiamonds not only hold important promise for practical use, but also likely play an important role in star formation and the evolution of interstellar matter due to their remarkable stability.^7^ They have been detected in large abundances in meteorites^8^, ^9^, ^10^ and it has been suggested that they are responsible for infrared emission features near 3.43 and 3.53 μm observed towards a few astronomical sources^11^ as well as anomalous microwave emission.^12^ Based on astronomical infrared absorption bands, diamondoids have also been hypothesized to occur in interstellar ices.^13^ The emission bands that are hypothesized to be due to nanodiamonds are only observed towards a very small number of astronomical sources, which was attributed to their inefficient optical excitation as a consequence of the low oscillator strengths of the electronic transitions.^14^ This contrasts with polyaromatic hydrocarbons (PAHs) that typically exhibit strong electronic transitions, which moreover fall at longer wavelengths, causing their interstellar IR emission bands to be ubiquitous and intense.^15^, ^16^, ^17^ Estimates suggest that diamondoids may comprise 1–3 % of the total cosmic carbon budget.^14^

Sparked by the astrophysical relevance, a large number of experimental and computational studies have been devoted to understanding the spectroscopic properties of neutral and ionized diamondoids in the gas phase. The ionization potentials of the first five members of the diamondoid family and the electronic structure of a series of diamondoids have been investigated by valence photoelectron spectroscopy.^18^, ^19^ Laser‐ and synchrotron‐induced vibrationally resolved photoluminescence spectra of diamondoids isolated in the gas phase have also been reported.^20^, ^21^ Infrared and Raman spectra of neutral and cationic diamondoids have been measured and compared with Density Functional Theory (DFT) computed spectra.^22^, ^23^, ^24^ Electronic spectra and ionization energies have also been characterized by DFT together with infrared spectra of neutral and cationic diamondoids up to C_38_H_42_.^14^ Infrared emission spectra of hot diamondoids have been recorded, providing additional evidence for the assignments of the interstellar IR emission bands to diamondoids of a few nanometers in size.^25^ Adamantane is the smallest member of the diamondoid family and can be considered to be the basic building block consisting of a single diamond cage. IR spectra have been reported for adamantane^26^, ^27^, ^28^ and its radical cation, the latter undergoing Jahn‐Teller distortion, lowering the symmetry to from T_d_ to C_3v_.^29^

In an interstellar environment, nanodiamonds are subject to strong energetic processing, such as irradiation by strong interstellar radiation fields, and chemical transformations including unimolecular dissociations may occur. The dissociation of adamantane (C_10_H_16_) has been studied in some detail, both experimentally and computationally. The closed‐shell product formed by hydrogen atom loss from the adamantane radical cation, the adamantyl cation (C_10_H_15_^+^), was spectroscopically characterized in the gas phase.^30^ Later, vacuum ultraviolet (VUV) dissociation of small diamondoids trapped in a cryogenic inert matrix was studied.^31^ The fragmentation of adamantane by electron ionization (EI) and proton transfer ionization (PTI) have been studied by mass spectrometry and it was suggested that the cage opens up to form aromatic species.^32^—^34^ Very recently, the gas phase dissociation of adamantane was studied using a combination of Vacuum Ultraviolet (VUV) single photon dissociative ionization measurements and quantum‐chemical computations.^35^ Based on potential energy surface (PES) calculations, structures were suggested for the dissociation products and accurate appearance energies were derived from a statistical model.

In this study, we employ mass spectrometry‐based infrared multiple‐photon dissociation (IRMPD) spectroscopy to characterize the molecular structure of the products formed in the dissociative ionization of adamantane. This methodology was successfully applied to structurally identify the product ions in ion chemistry studies, including those from resonance‐enhanced two‐photon (193 nm) dissociative ionization of (nitrogen‐containing) aromatic hydrocarbons.^36^, ^37^ Here, in absence of an intermediate electronic state in adamantane, we employ electron ionization to generate the ion and its fragments, which are subsequently mass isolated and interrogated by IR action spectroscopy.

## 2 Methods

### 2.1 Experimental

Experiments were carried out on a quadrupole ion trap time‐of‐flight (QIT‐TOF) mass spectrometer coupled to the beamline of the IR free electron laser FELIX as described in detail elsewhere.^36^, ^37^, ^38^ Briefly, a radio frequency (RF, 1 MHz) Paul‐type QIT is mounted inside a vacuum chamber at a pressure of <10^−7^ mbar. Helium is supplied to the ion trap raising the pressure to 5×10^−5^ mbar to thermalize and focus the ions to the center of the trap, improving the mass resolution of the mass spectrometer. Furthermore, the reduced size of the ion cloud improves overlap with the focused free electron laser beam.

Adamantane (Sigma Aldrich, C_10_H_16_, 99 %) vapor is introduced effusively into the vacuum chamber using a dosing valve. An electron ionization source is located in front of one of the endcap electrodes of the QIT. Electrons with an energy of 70 eV (dissociatively) ionize the adamantane vapor. The ions are pushed towards a hole in the endcap and two gate electrodes just before the endcap admit the ions to the QIT during the trap filling stage at the beginning of the measurement cycle. Mass isolation of the (fragment) ion of interest is achieved by an 80‐ms long Stored Waveform Inverse Fourier Transform (SWIFT) pulse,^39^ which resonantly excites the axial secular frequencies of ions that are to be rejected from the trap.

Mass analysis is accomplished by pulse‐extracting the ions from the QIT through a hole in the extractor endcap. Ions are accelerated by an extraction grid at −2375 V and enter the 50 cm long TOF tube. The flight tube liner and the front grid of the detector are at the same potential as the extraction grid to create a field‐free region. Ions are detected on a Z‐gap type microchannel plate (MCP) detector. The resulting mass resolution (m/Δm) is better than 200.

Infrared spectra of the isolated ions are recorded through IRMPD spectroscopy^38^,^40^ using the intense radiation from the free electron laser for infrared experiments (FELIX),^41^ which was set up to produce radiation in the wavelength range from 5.5 to 16.7 μm (1818–600 cm^−1^). Macropulse energies ranged from 50 to 70 mJ at a relative bandwidth of about 0.5 %. The IR beam is tightly focused onto the ion cloud in the center of the QIT. The photon energy is tuned from 1800 to 600 cm^−1^ in steps of 3 cm^−1^ and 40 mass spectra are averaged at each photon energy. The IR spectrum is constructed by plotting the fragment yield of the isolated ion as a function of wavelength. Dissociation in all reported IRMPD spectra is facile and reaches maximum values of 75–90 %, suggesting that the main isomers of the ion population are probed. Spectra are linearly scaled to account for variations in the IR laser pulse energy.

### 2.2 Computational

Vibrational spectra of candidate molecular structures of each of the adamantane fragment ions were predicted by density functional theory computations using the Gaussian09 software package.^42^ Structures are optimized at the B3LYP/6‐311G(d,p) level of theory and harmonic frequencies are computed and scaled by a uniform factor of 0.96 to empirically account for anharmonicities. The resulting stick spectra are convoluted with a Gaussian lineshape function with a Full‐Width‐at‐Half‐Maximum (FWHM) of 30 cm^−1^ to facilitate convenient comparison with the experimental spectra. Relative energies of isomeric species are determined from their ZPE corrected energies.

## 3 Results and Discussion

**Figure 1:**
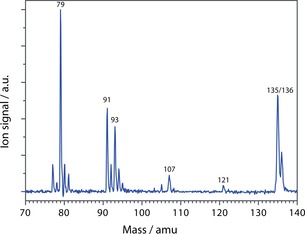
Time‐of‐Flight mass spectrum of the dissociative ionization of adamantane using an electron energy of 70 eV. The adamantane radical cation (C_10_H_16_^.+^) corresponds to the signal at m/z 136.

Adamantane was ionized using 70 eV electrons giving the mass spectrum shown in Figure 1, which is very similar to that reported in the NIST database.^43^ Observed fragment masses are listed together with their relative integrated intensities in Table 1. The strongest ion signals are found at *m/z* 77, 79, 80, 81, 91, 92, 93, 94, 107 and 135. These products do not necessarily form in a single‐step unimolecular dissociation, but may also form by multistep dissociation. This is particularly clear for the formation of the C_7_H_7_^+^ and C_6_H_5_^+^ ions that cannot be associated with a single neutral fragment. The fragments at *m/z* 79, 93 and 107 were individually mass‐isolated in the trap and their IRMPD spectra were recorded to derive their molecular structures, as detailed below.

**Table 1:** List of observed EI fragment mass‐to‐charge ratios, corresponding products ions and relative intensities of the product ions formed by dissociative ionization of adamantane.

m/z	Product ion	Relative intensity
136	C_10_H_16_^.+^	0.10
135	C_10_H_15_^+^	0.25
121	C_9_H_13_^+^	0.01
107	C_8_H_11_^+^	0.03
94	C_7_H_10_^.+^	0.03
93	C_7_H_9_^+^	0.10
92	C_7_H_8_^.+^	0.03
91	C_7_H_7_^+^	0.13
81	C_6_H_9_^+^	0.02
80	C_6_H_8_^.+^	0.03
79	C_6_H_7_^+^	0.23
77	C_6_H_5_^+^	0.04

### 3.1 The *m/z* 79 Ion

**Figure 2:**
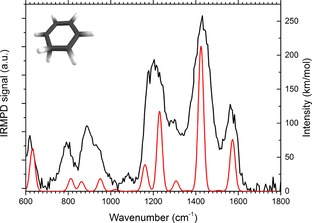
IRMPD spectrum of the isolated m/z 79 ion formed in the dissociative ionization of adamantane shown together with the DFT computed spectrum of protonated benzene.

Electron ionization of adamantane produces a prominent mass peak at *m/z* 79 (see Figure 1). An obvious candidate structure for this fragment is protonated benzene, but alternative structures are not *a priori* impossible. Therefore, an IRMPD spectrum was recorded for this ion. IR induced dissociation of this fragment ion yields only a single product at *m/z* 77, presumably by H_2_ loss. The IRMPD spectrum of *m/z* 79 is shown in Figure 2 together with the computed spectrum of protonated benzene and the match between the measured and computed spectrum is convincing. Other isomers such as 1‐ and 2‐protonated fulvene (at +39.3 and +108.7 kJ/mol relative to protonated benzene, respectively) and the methyl‐substituted cyclopentadienyl cation (+137.1 kJ/mol) do not match the recorded spectrum (see SI Figure S1). The *m/z* 79 fragment ion can thus be securely identified as protonated benzene.

Molecular hydrogen loss from protonated benzene (arenium ion) formed from various precursors has been studied in much detail in the past. From mass‐analyzed ion kinetic energy spectrometry (MIKES) measurements it was concluded that the energy release is in accord with a 1,1‐elimination from a σ‐complex and that proton scrambling occurs before H_2_ is expelled.^44^, ^45^ Later, the infrared spectrum was also recorded and it was confirmed that the σ‐complex, which represents the global minimum on the potential energy surface, is indeed the dominant species.^46^, ^47^, ^48^

The finding that the arenium ion is formed can also be compared with results for VUV induced dissociation reported recently by Candian et al.^35^ VUV dissociation was found to mostly result in a radical‐cation C_6_H_8_^.+^ fragment with the even‐electron C_6_H_7_^+^ ion being a weaker contributor. Based on quantum‐chemical computations, the radical fragment of C_6_H_8_^.+^ composition is suggested to be the radical cation of 1,3‐cyclohexadiene. Our EI mass spectrum features hardly any C_6_H_8_^.+^ (see Figure 1) highlighting clear differences between EI and VUV dissociative ionization processes.

### 3.2 The *m/z* 93 Ion

**Figure 3:**
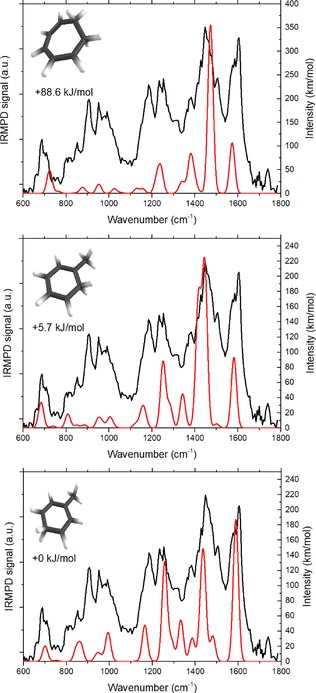
IRMPD spectrum (black) of the isolated m/z 93 ion displayed together with DFT computed spectra (red) of the 1,2‐dihydrotropylium cation (top), ortho‐protonated toluene (middle) and para‐protonated toluene (bottom).

The IR spectrum of the fragment ion at *m/z* 93 (C_7_H_9_^+^) is derived from the IRMPD‐induced signals into *m/z* 91, 79 and 77. Conceivable structures for the C_7_H_9_^+^ ion include protonated toluenes, dihydrotropylium ions, and protonated isotoluenes. A comparison of the experimental spectrum with predicted spectra for a comprehensive set of these isomers is shown in Supporting Information Figures S2 through S4. The best spectral matches are found with the computed spectra for *ortho*‐protonated toluene (*o*‐pt), *para*‐protonated toluene (*p*‐pt) and 1,2‐dihydrotropylium (1,2‐dht), which are summarized in Figure 3.

The computed spectra of *o*‐pt and *p*‐pt match the experimental spectrum better than the computed spectrum of 1,2‐dht does, but the latter cannot be excluded as contributing isomer. *o*‐pt is computed to be 5.7 kJ/mol higher in energy than the *para* isomer, which is the lowest energy isomer considered here. The 1,2‐dht isomer is the lowest energy isomer among the dihydrotropylium ions, but lies at a large energy gap of 88.6 kJ/mol from the ground state *p*‐pt. Significant contributions by other protonated non‐aromatic isomers are unlikely, as these are thermodynamically far more unfavorable. The suggestion that *o*‐pt is a likely contributor to the spectrum is in line with the extensive potential energy surface computations that suggest *o*‐pt to be the product ion formed over the lowest‐lying transition state, but the other isomers cannot be ruled out.^35^

Extensive studies on the structure and dissociation mechanism of C_7_H_9_^+^ ions formed from various precursors have been reported in the literature. It was shown that hydrogen scrambling occurs as well as scrambling of carbon atoms via a ring expansion (to protonated cycloheptatriene) and subsequent ring contraction back to toluenium.^33^,^45^,^49^, ^50^, ^51^ Our infrared spectroscopic assignment can best be compared with a mass spectrometric gas‐phase titration study of C_7_H_9_^+^ ions formed by electron ionization of two monoterpenes, α‐pinene and limonene (two C_10_H_16_ isomers of adamantane).^33^ From this work it was concluded that more than 85 % of the C_7_H_9_^+^ ions exists in the form of toluenium ions, with a very small fraction (<10 %) of protonated dihydrotropylium ions. The observation that our *m/z* 93 spectrum best resembles the computed spectra of protonated toluene, while at the same time not excluding contributions of the dihydrotropylium ion is in good agreement with this study and also with the spectroscopically confirmed most favorable protonation sites being the *ortho* and *para* positions.^48^,^52^ However, the detection of the weak *m/z* 79 signal that results from IRMPD in our experiment deviates from the CID results and from an IRMPD spectroscopic study on the toluenium ion.^53^ The *m/z* 79 fragment possibly results from CH_2_ loss from the parent ion and could be indicative of a small fraction of the C_7_H_9_^+^ ions existing in the form of protonated isotoluenes, which to date have not been identified as products in the C_7_H_9_^+^ manifold.^33^,^44^, ^45^,^49^ However, the signal is too weak to confirm this assignment spectroscopically accounting for less than 10 % of the ion population.

### 3.3 The *m/z* 107 Ion

**Figure 4:**
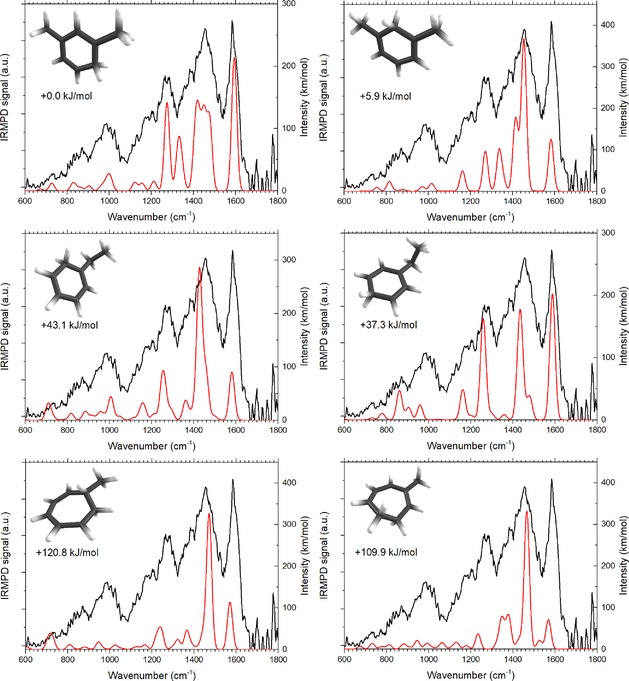
IRMPD spectrum (black) of the isolated fragment ion at m/z 107 together with the computed spectra (red) of (top) 4‐protonated m‐xylene and 2‐protonated m‐xylene and (middle) 2‐protonated ethylbenzene and 4‐protonated ethylbenzene, and (bottom) 1‐protonated 1‐methyl‐1,3,5‐cycloheptatriene and 6‐protonated 3‐methyl‐1,3,5‐cycloheptatriene. Relative free energies at 0 K of the different isomers are indicated.

Figure 4 displays the IRMPD spectrum of the *m/z* 107 ion with C_8_H_11_^+^ composition that is formed by electron ionization of adamantane. Wavelength‐dependent IR dissociation of isolated *m/z* 107 ions yields mass channels 105, 91, 79, and 77, which were used to construct the IRMPD spectrum of C_8_H_11_^+^. A vast number of isomers is obviously conceivable for this composition, and we restrict ourselves here to those that have been reported in mass‐spectrometric studies.^49^,^54^, ^55^ We consider the various protonated forms of ethylbenzene, xylene, vinylcyclohexadiene and methylcycloheptatriene as possible structures for the C_8_H_11_^+^ fragment. For the protonated xylenes we assume that the tertiary carbons of adamantane remain in their original relative position, giving the various protomers of *m*‐xylene (1,3‐dimethylbenzenes).

The experimental spectrum is compared with computed spectra for a large number of potential isomers in Supporting Information Figures S5–S10. The closest spectral matches are found for the computed spectra of 2‐ and 4*‐*protonated ethylbenzene (peb), and 2‐ and 4‐protonated *m‐*xylene (pmx), which are overlaid onto the experimental spectrum in Figure 4. The spectra of two of the protonated methylcycloheptatriene isomers do not conflict with the measured spectrum as shown in the bottom panel of Figure 4. Computed spectra for the various protonated vinylcyclohexadiene isomers shown in the Supporting Information do not agree well with the measured spectrum. Based on the comparison in Figure 4 it is not possible to make a single unique assignment and multiple isomers can contribute to the measured spectrum. Relative energies may to some extent guide our choice of isomers. The two xylene isomers are lowest in energy with relative energies of 0 kJ/mol (4‐pmx) and +5.9 kJ/mol (2‐pmx). The *o*‐ and *p‐*peb cations are calculated to be the two lowest‐energy ethylbenzene cations (see SI), with protonation in the *para* position at +37.3 kJ/mol being 5.8 kJ/mol more favorable than protonation at the *ortho*‐position. The methylcycloheptatriene isomers are much higher in energy, with 6‐protonated 3‐methyl‐1,3,5‐cycloheptatriene being lower in energy at 109.9 kJ/mol and 1‐protonated 1‐methyl‐1,3,5‐cycloheptatriene at 120.8 kJ/mol, and are thus less likely contributors to the spectrum based on energetics arguments.

The dissociation of the various C_8_H_11_^+^ isomers has been investigated using various mass‐spectrometric techniques.^49^,^54^, ^55^, ^56^ Dissociation of protonated *ortho*‐ and *meta*‐xylenium ions was found to yield mostly CH_4_‐loss with a smaller fraction of H_2_‐loss, while that of the ethylbenzenium ions yields mostly loss of ethene^49^ and this was found to be in agreement with the C_8_H_11_^+^ potential energy surface.^55^ The most prominent ions produced by IRMPD are [M−CH_4_]^+^ and [M−C_2_H_4_]^+^ with traces being detected at *m/z* 105 and 77, the latter likely formed by secondary hydrogen loss from [M−C_2_H_4_]^+^. These dissociation products are largely in line with the suggestion of protonated ethylbenzenes and protonated xylenes being formed from EI induced dissociation of adamantane. The C_8_H_11_^+^ fragment ion was not reported in a recent VUV dissociative ionization study of adamantane.^35^ Instead, VUV ionization produces an ion of C_8_H_12_^.+^ composition. The associated PES calculations suggest this ion to be identified as 4‐ethylidenecyclohex‐1‐ene, which is indeed analogous to the ethylbenzenes that provide a reasonable match with the IR spectrum for the C_8_H_11_^+^ fragment reported here.

### 3.4 Other Fragments: *m/z* 135 and 91

Dissociative ionization of adamantane gives a strong mass peak at *m/z* 135 due to the loss of a single H‐atom. The infrared spectrum of this fragment ion has been investigated previously using chemical ionization (CI) and IRMPD spectroscopy.^30^ The ion was structurally assigned as the 1‐adamantyl cation formed by H‐loss from one of the tertiary C‐atoms. The 2‐adamantyl cation, which would be formed via H‐loss from one of the secondary C‐atoms, was excluded. The spectrum of the adamantyl cation generated by EI reported here is shown in Supporting Information Figure S17 and identifies the 1‐adamantyl cation as the product ion structure analogous to the results in Ref. [30]. The relative energy of the 1‐adamantyl cation is calculated to be 44.7 kJ/mol lower than that of the 2‐adamantyl cation.

The fragment ion at *m/z* 91 is one of the most abundant products of dissociative ionization of adamantane (see Figure 1). Upon mass isolation and IR irradiation, we do not observe dissociation at any IR wavelength and therefore no experimental IR spectrum could be obtained for this product ion. The nominal mass of 91 corresponds to a cation with C_7_H_7_ composition, which likely corresponds to either the benzylium or the tropylium ion, or a mixture thereof. The lack of dissociation can be explained by the high stability of these systems. Formation and differentiation of these species in spectroscopic and MS experiments has been widely discussed in the literature, as the *m/z* 91 peak is prominent in many MS studies on hydrocarbon species.^57^, ^58^, ^59^, ^60^ The branching between the two isomers in our experiments could be derived from the ion‐molecule reaction with neutral toluene, in which only the benzylium isomer is converted while the tropylium ion is unreactive.^61^ Such an analysis has not been conducted here.

## 4 Concluding Remarks

Infrared spectra were recorded for the main ionic fragments formed upon dissociative electron ionization of adamantane. In combination with quantum‐chemically predicted vibrational spectra for potential structures, this has enabled us to establish molecular structures for the major ionic fragments at *m/z* 79 and 135. For other dissociation products we have narrowed down the list of potential contributors to the spectrum.

**Figure 5:**
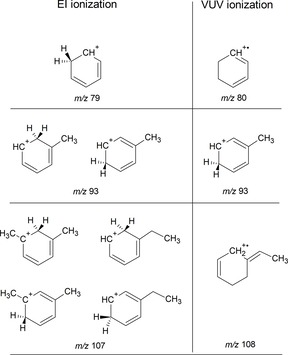
Structures of the product ions from electron ionization of adamantane as suggested by mid‐IR action spectroscopy (left) compared to the main ionic fragments found in VUV dissociative ionization as characterized by DFT potential energy surface computations.^35^

An overview of the structures identified as the main product ions in this work is given in Figure 5. A comparison is made with the main products detected in VUV‐induced dissociative ionization, where structural identification was done on the basis of extensive PES computations.^35^ The observed fragmentation pathways are clearly different, which is qualitatively not surprising as the underlying processes are very different in terms of energy deposited, selection rules involved and potential multistep dissociation reactions. One notices that the main products from EI ionization are in all cases even‐electron species. This may suggest that the main dissociation pathways traverse the closed‐shell adamantyl cation that is readily formed from adamantane,^62^ and from there follow the “even‐electron rule” common to organic mass spectrometry.^63^ In contrast, the main product ions from VUV‐induced dissociation comprise both even‐ and odd‐electron ions.^35^ The two processes therefore appear to be fundamentally different.

EI induced fragmentation furnishes the *m/z* 79 ion (protonated benzene), while VUV dissociation yields the cyclohexadiene (*m/z* 80) ion. The IR spectrum of the *m/z* 93 species formed from electron ionization of adamantane agrees with the C_7_H_9_^+^ products formed by electron ionization of two C_10_H_16_ isomers, α‐pinene and limonene, characterized by gas‐phase titration.^33^ Furthermore, this finding is also in agreement with extensive C_10_H_16_^.+^ PES computations that suggest that the formation of toluenium is kinetically favored.^35^ Identification of the *m/z* 107 ion is perhaps most challenging in the present study; it is difficult to make a unique assignment based on the IR spectra, but two ethylbenzene and two *meta*‐xylene structures are likely contributors to the ion population, while two protonated methylcycloheptatrienes isomers cannot be ruled out. Other isomers are however unlikely contributors, because their spectra conflict with the measured *m/z* 107 spectrum. The ethylbenzenes appear to be analogs of the 4‐ethylidenecyclohexene that is predicted by the DFT calculations for the *m/z* 108 fragment ion observed in VUV dissociative ionization.

We conclude with the more general notion that it has been very challenging to understand EI mass spectra of organic molecules, let alone to predict them from first principles.^64^ The ability to record IR spectra for mass‐selected fragment ions provides an elegant way to increase our understanding of the electron ionization of organic molecules. The ions produced in the dissociation of adamantane are commonly detected EI products of alkylbenzenes, which have been widely discussed in physical‐organic mass spectrometry studies. We have shown that the presence of both 6‐ and 7‐membered ring C_7_H_9_^+^ isomers in our EI mass spectrum is in harmony with EI mass spectrometry and collision‐induced dissociation experiments of alkylbenzenes. Some of the detected product ions can probably not be assigned to a single isomer, but rather to a mixture of species.

From the IR spectral data reported here, as well as from the VUV dissociative ionization study,^35^ it becomes clear that an efficient conversion from a purely aliphatic *sp* ^*3*^ structure to aromatic systems occurs in the gas‐phase dissociation of the adamantane cation. This suggests that there exist facile chemical pathways from diamondoid species to aromatic molecules, which may also be operative in interstellar and circumstellar environments. Based on mass‐spectrometric studies, it has been hypothesized that the next diamondoid in the series, diamantane, yields branched benzenes rather than functionalized naphthalenes.^32^ Spectroscopic studies on the dissociation products of these larger, perhaps more astronomically relevant, diamondoids are required to more firmly establish the astrochemical link between diamondoids and (poly‐)aromatic species.

## Conflict of interest

The authors declare no conflict of interest.

## Supporting information

As a service to our authors and readers, this journal provides supporting information supplied by the authors. Such materials are peer reviewed and may be re‐organized for online delivery, but are not copy‐edited or typeset. Technical support issues arising from supporting information (other than missing files) should be addressed to the authors.

Supplementary
