# Supplementary material for: Spectroscopic Characterization of the Product Ions Formed by Electron Ionization of Adamantane
Source: Chemphyschem. 2018 Oct 30;19(23):3211–8. doi: 10.1002/cphc.201800846 (PMC6392131; doi:10.1002/cphc.201800846)
Supplement: Supplementary file 1 — Supplementary [file CPHC-19-3211-s001.pdf]

Supporting Information

© Copyright Wiley-VCH Verlag GmbH & Co. KGaA, 69451 Weinheim, 2018

Spectroscopic Characterization of the Product Ions Formed by Electron Ionization of Adamantane

Jordy Bouwman,* Stefan Horst, and Jos Oomens© 2018 The Authors. Published by Wiley-VCH Verlag GmbH & Co. KGaA. This is an open access article under the terms of the Creative Commons Attribution License, which permits use, distribution and reproduction in any medium, provided the original work is properly cited.

Supporting information

In the main text of the manuscript, only the computed spectra of the best matching isomers are shown. Here, for completeness, a detailed overview showing comparison between measured IRMPD spectra and different isomers is presented.

m/z 79 fragment

The loss of a C_4H_9 fragment from the adamantane parent species results in an ion of C_6H_7 composition. The IRMPD spectrum recorded of the fragment is compared to the DFT computed spectra of various isomeric structures in Figure S1. A clear identification of protonated benzene can be made based on this comparison.

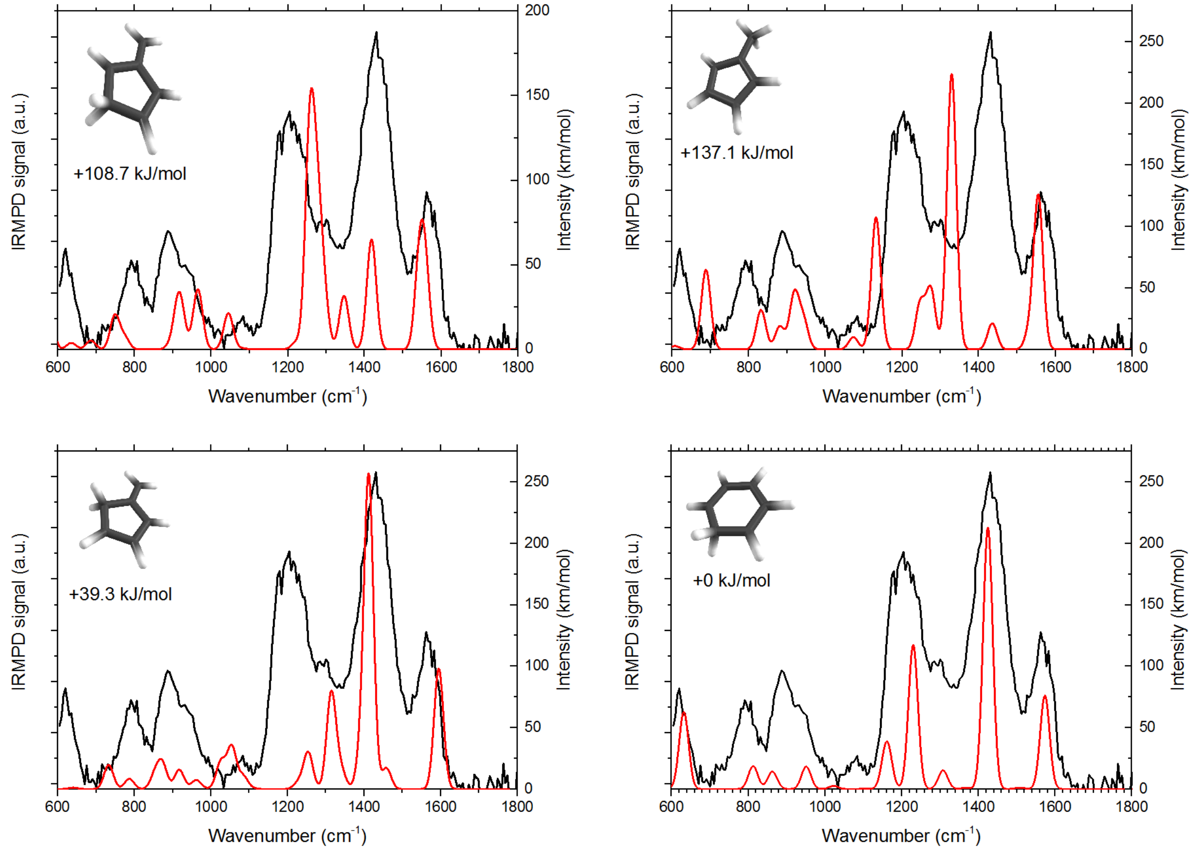

Figure S1 Measured IRMPD spectrum of the C_6H_7 cation compared to four isomers that possibly contribute.

m/z 93 fragment

The net loss of C_3H_6 from the adamantane precursor ion results in an ion of C_7H_9 composition. The IRMPD spectrum recorded for this fragment is compared to the DFT computed spectra of various isomeric structures in Figures S2-S4.

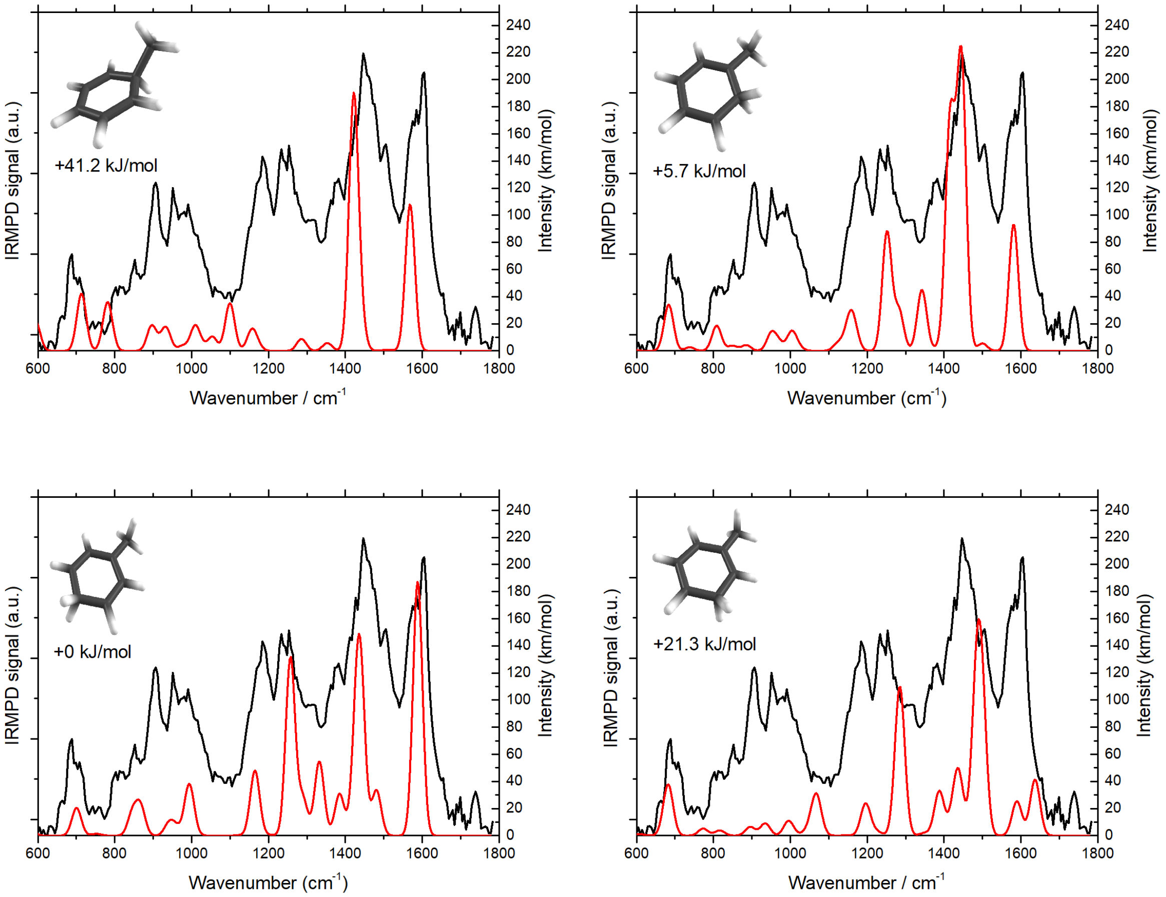

Figure S2: Measured IRMPD spectrum of the *m/z*=93 fragment generated by EI of adamantane compared to computed spectra of 1-, 2-, 3-, and 4-protonated toluene.

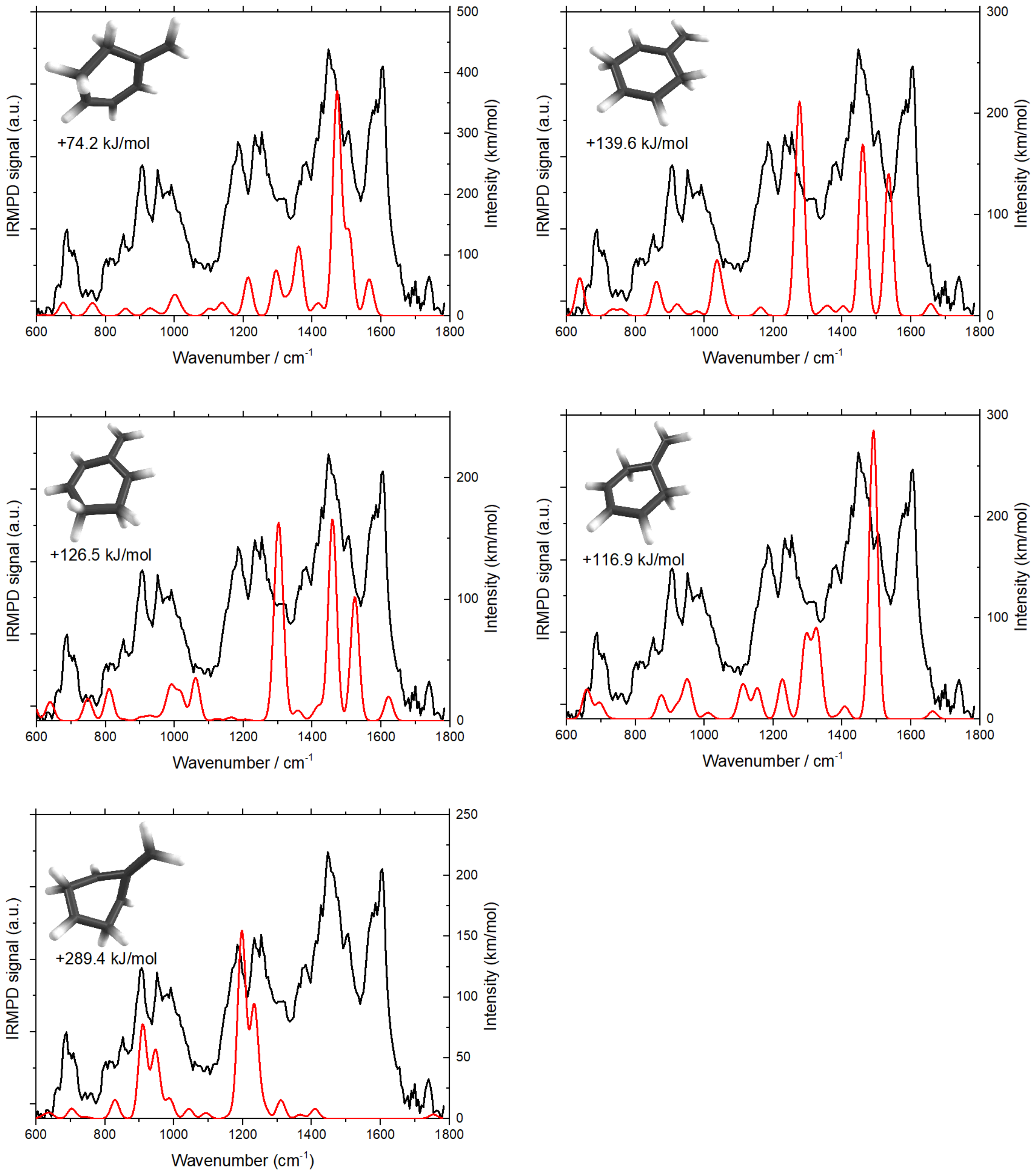

Figure S3: Measured IRMPD spectrum of the *m/z*=93 fragment generated by EI of adamantane compared to computed spectra of the various protonated isotoluenes.

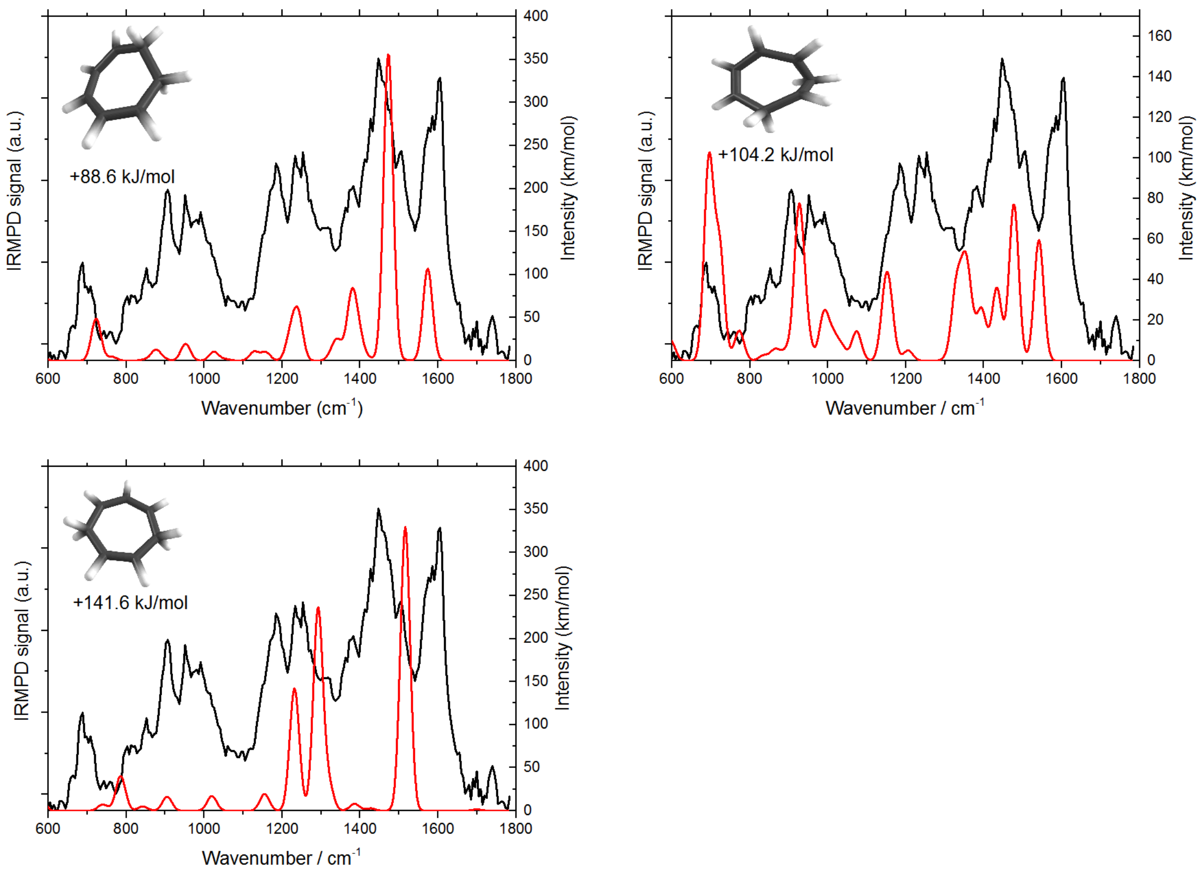

Figure S4: Measured IRMPD spectrum of the *m/z*=93 fragment generated by EI of adamantane compared to computed spectra of the various protonated cycloheptatrienes.

***m/z* 107 fragment**

A cation of C_8H_{11} composition is formed upon electron ionization of adamantane. The IRMPD spectrum recorded for this fragment is compared with the DFT computed spectra of various isomeric structures in Figures S5-S10.

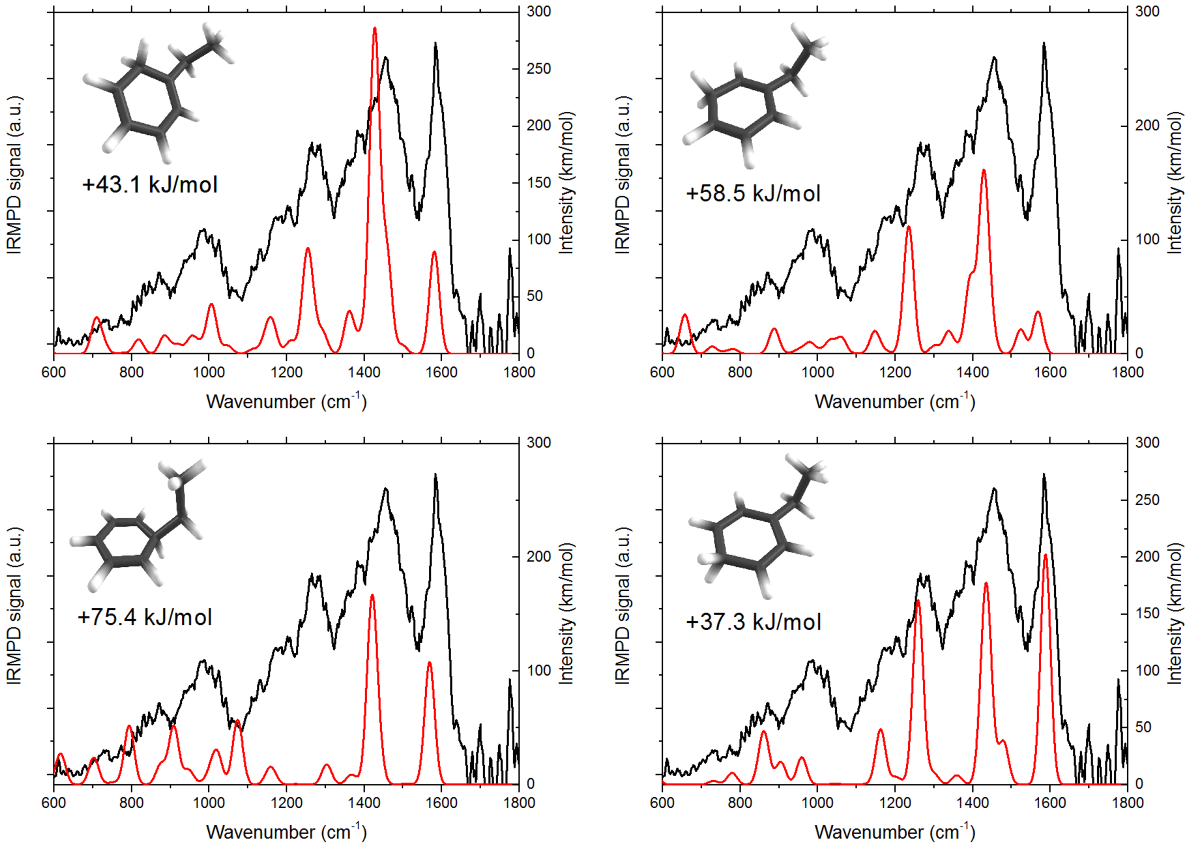

Figure S5: Experimental IRMPD spectrum of the m/z 107 ($C_8H_{11}^+$) fragment compared to computed spectra of the various isomers of protonated ethylbenzene.

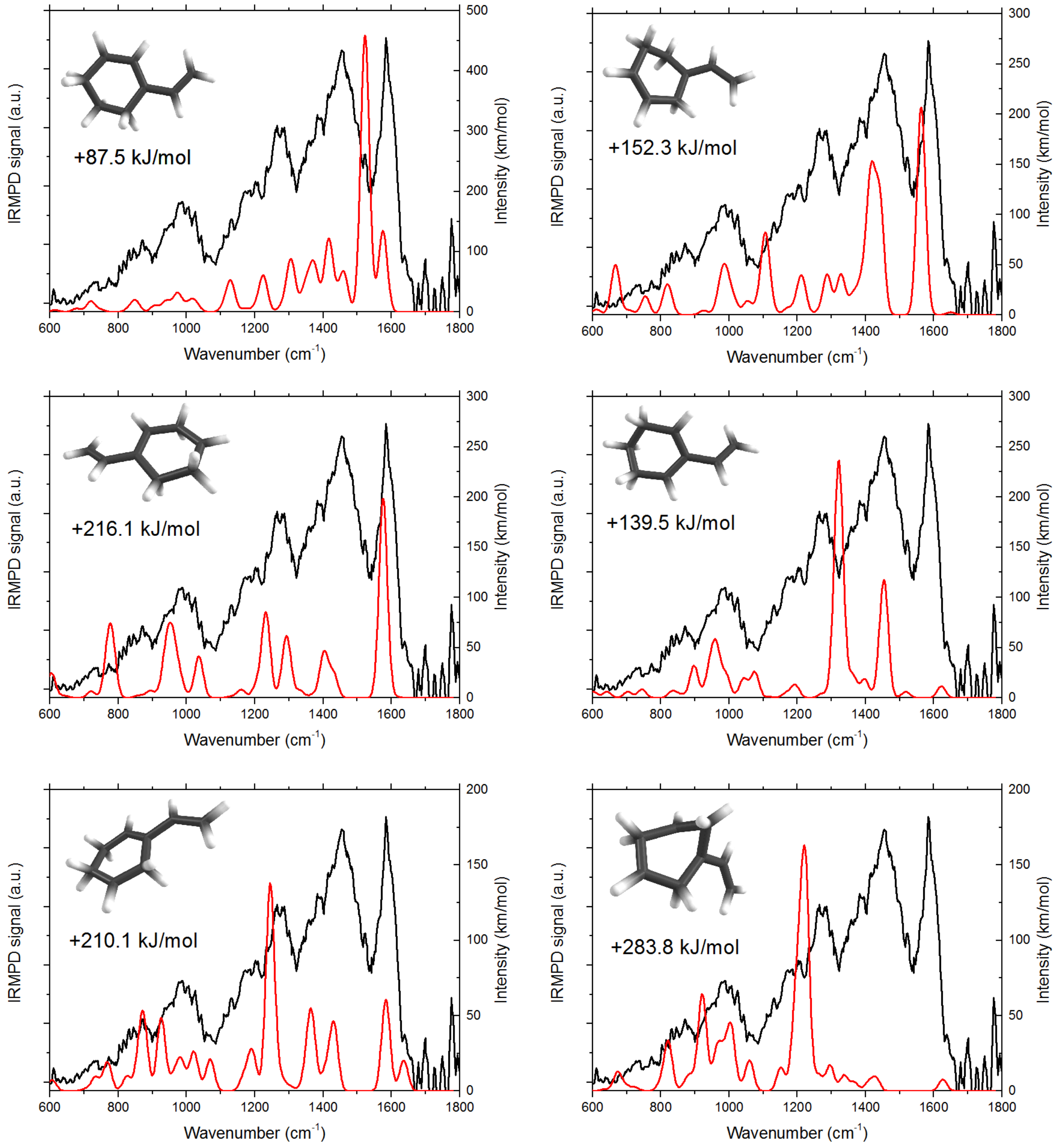

Figure S6: Experimental IRMPD spectrum of the m/z 107 ($C_8H_{11}^+$) fragment compared to computed spectra of various isomers of protonated vinylcyclohexadiene.

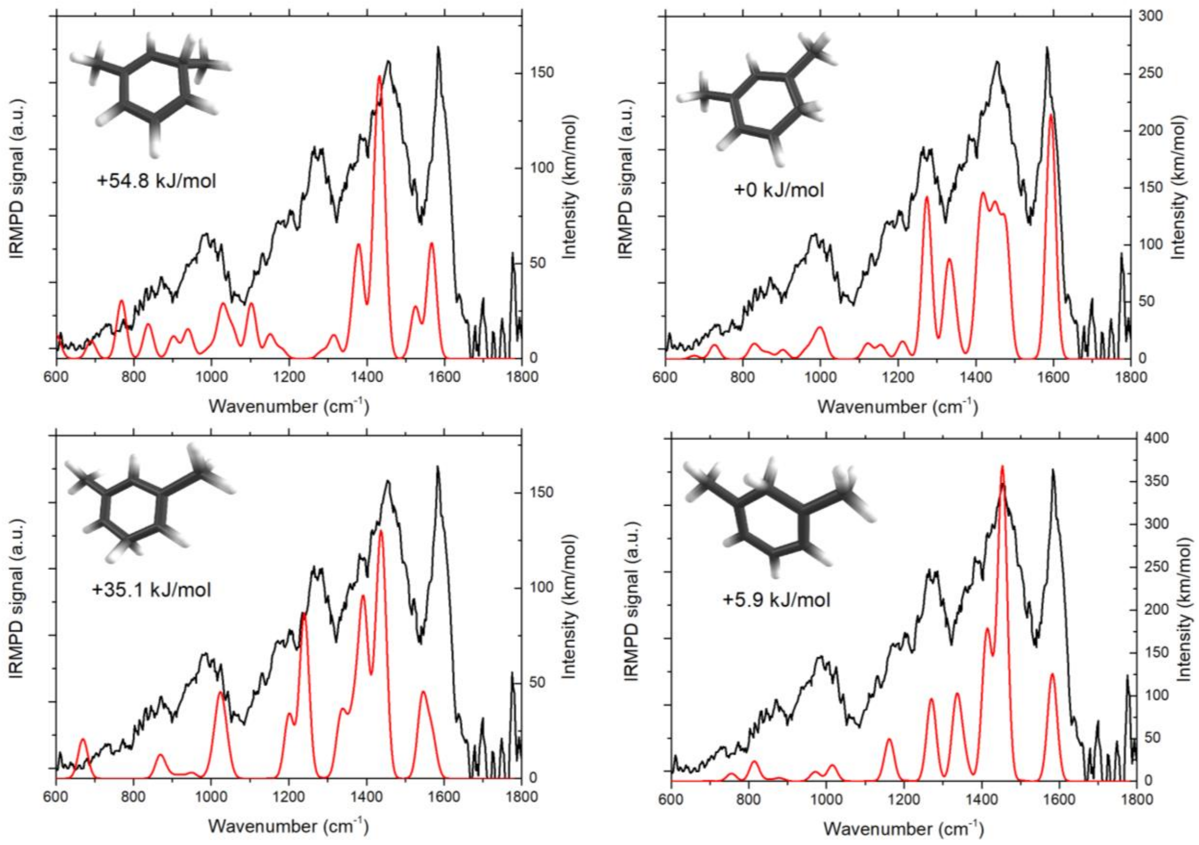

Figure S7: Experimental IRMPD spectrum of the m/z 107 ($C_8H_{11}^+$) fragment compared to computed spectra of the various isomers of protonated meta-xylene isomers.

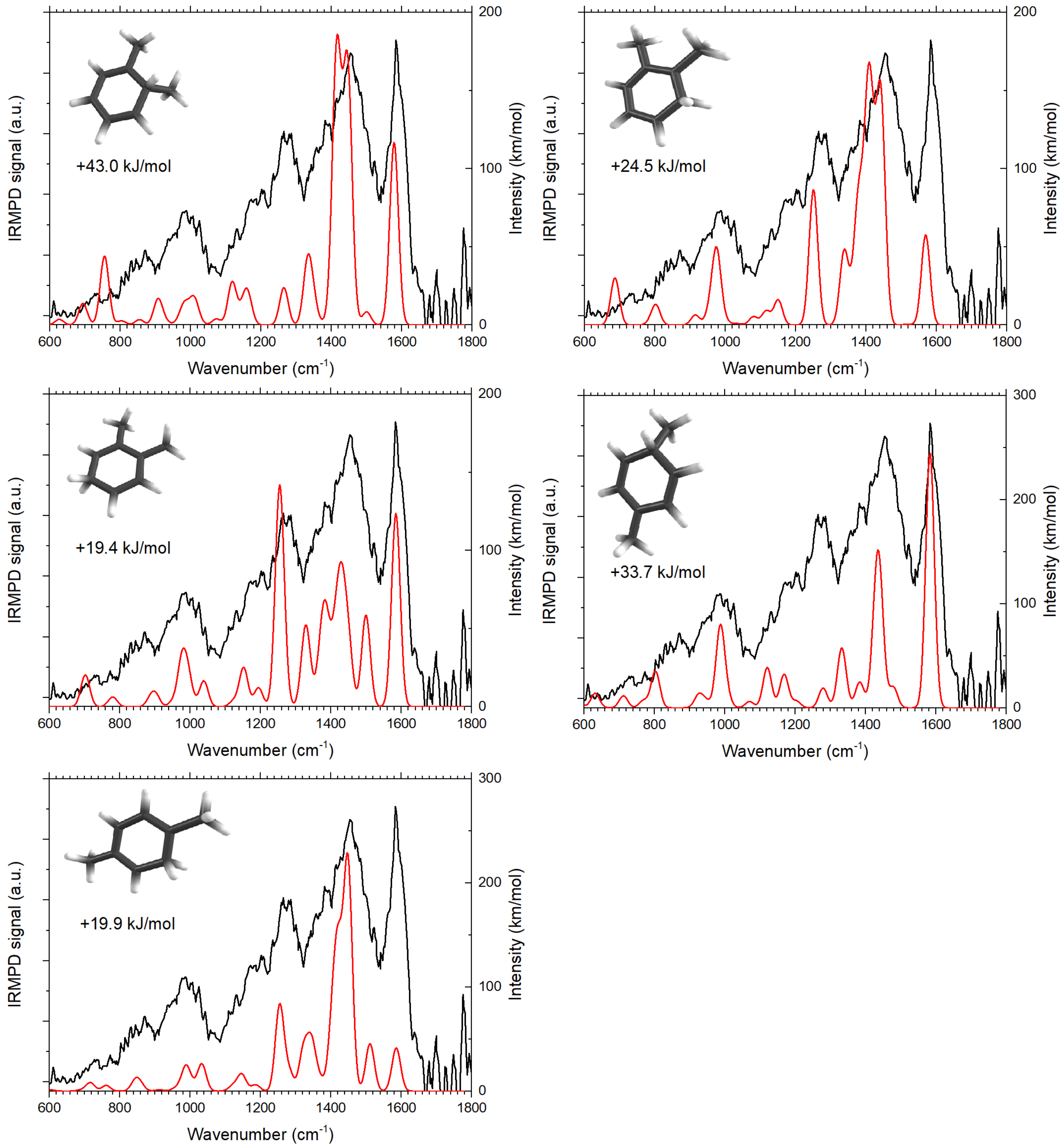

Figure S8: Experimental IRMPD spectrum of the m/z 107 ($C_8H_{11}^+$) fragment compared to computed spectra of the various isomers of protonated ortho- and para-xylene isomers.

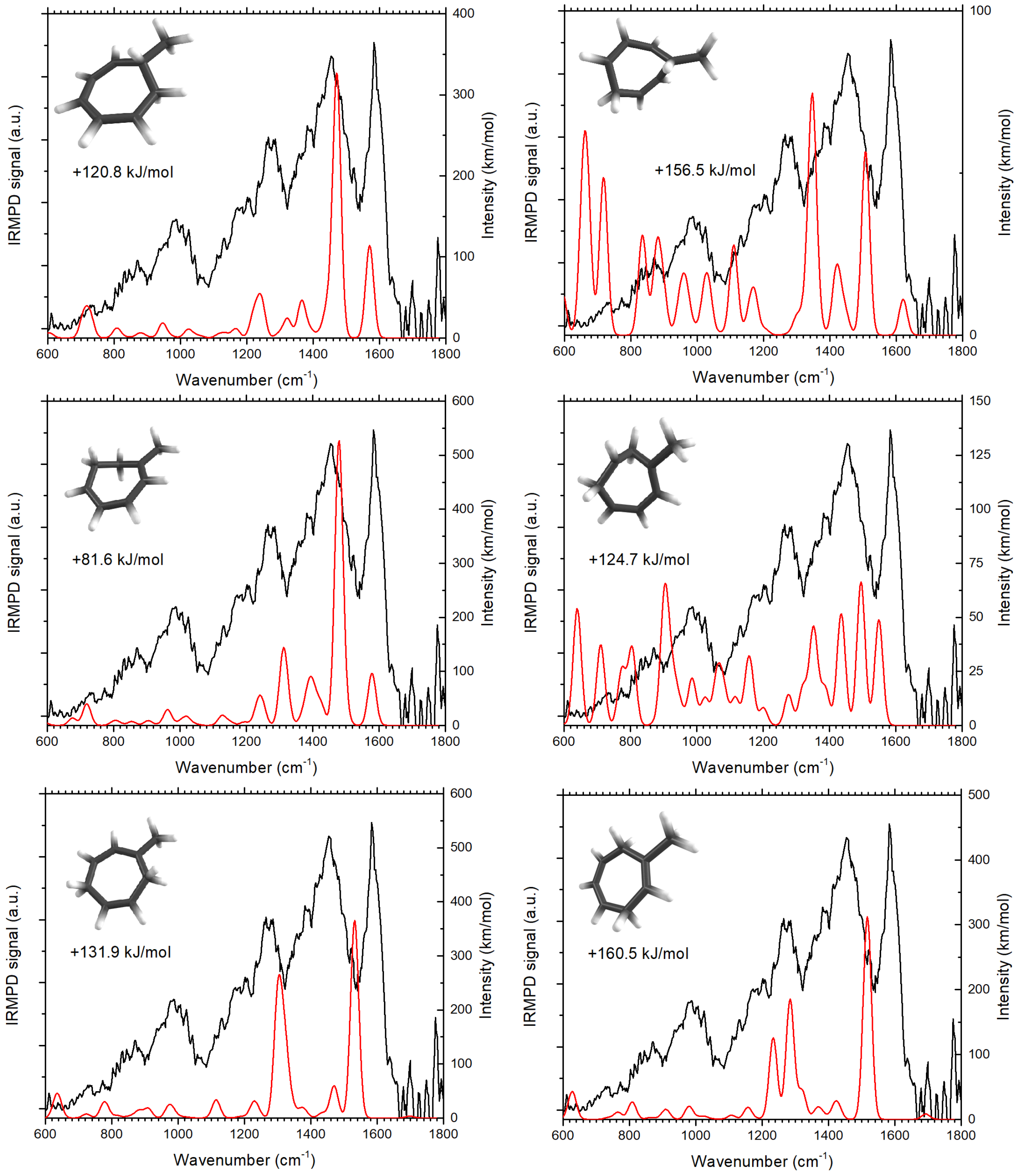

Figure S9: Experimental IRMPD spectrum of the m/z 107 ($C_8H_{11}^+$) fragment compared to computed spectra of the various isomers of protonated methylcycloheptatriene.

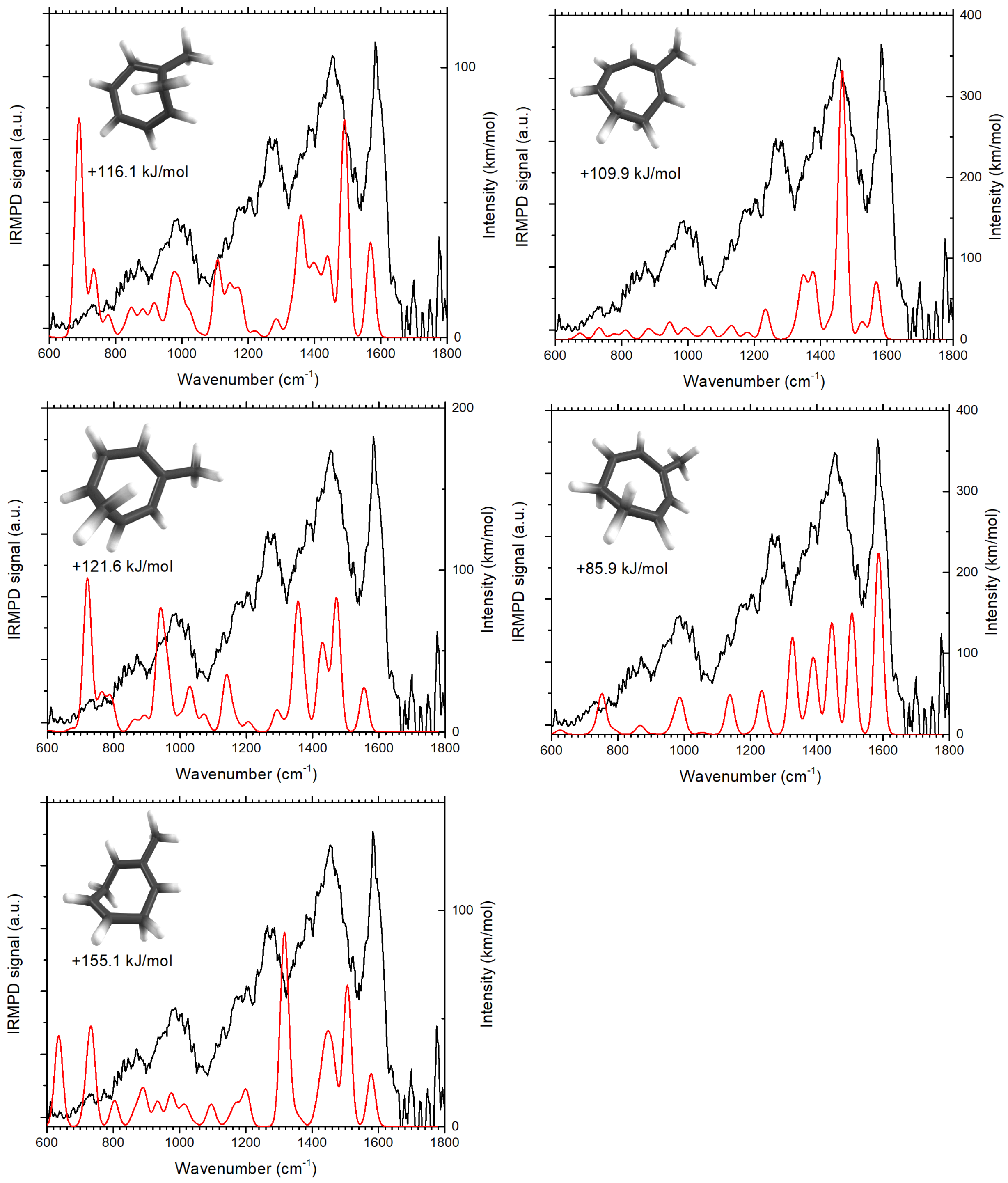

Figure S10: Experimental IRMPD spectrum of the m/z 107 ($C_8H_{11}^+$) fragment compared to computed spectra of the various isomers of protonated methylcycloheptatrienes. (Continuation of Figure S9)

m/z 121 fragment

Despite the very weak signal at *m/z* 121 in the mass spectrum, we have recorded a IRMPD spectrum of the $[M\text{-}CH_3]^+$ fragment. Many isomers can be responsible for this signal. The IRMPD spectrum recorded of the fragment is compared to the DFT computed spectra of various isomeric structures in Figures S11-S16. No unique isomer identification can be made based on these comparisons.

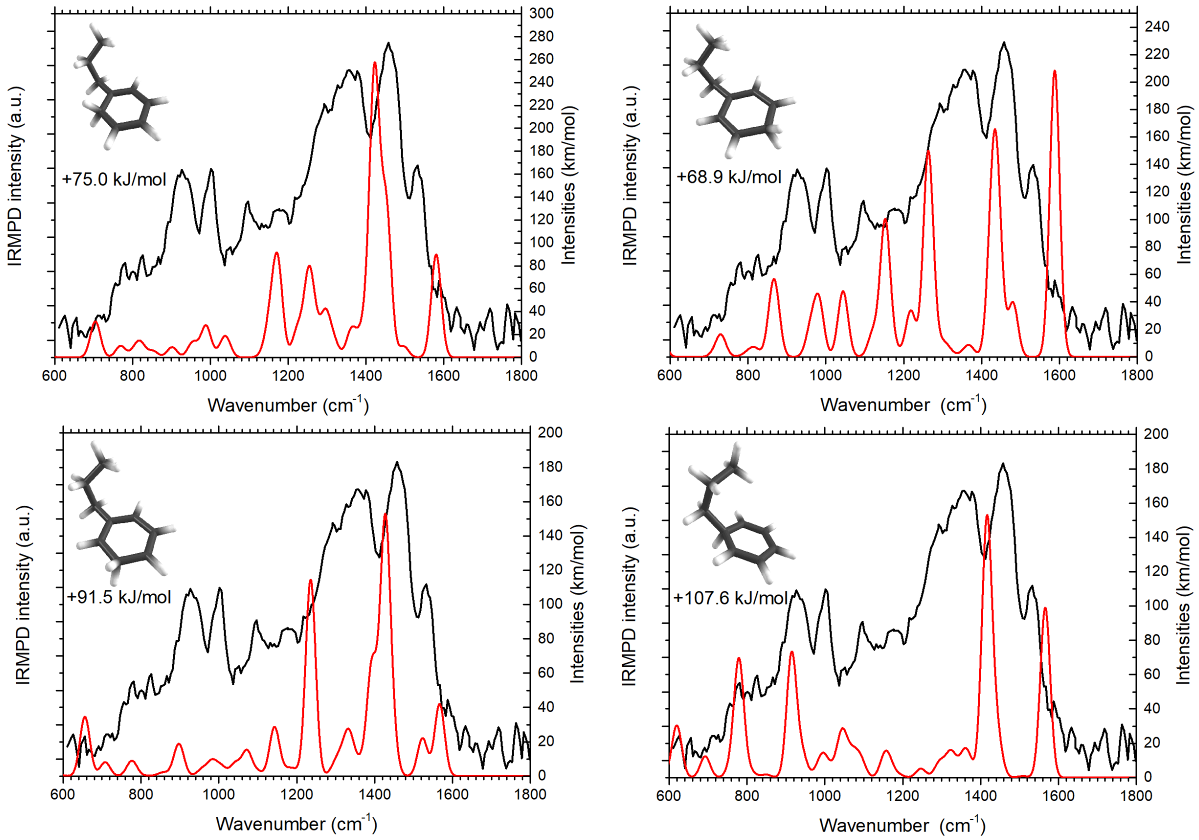

Figure S11: Spectrum of $[M\text{-}CH_3]^+$ formed upon EI of adamantane compared with the DFT computed spectra of the ipso-, ortho-, meta- and para-propylbenzene cation.

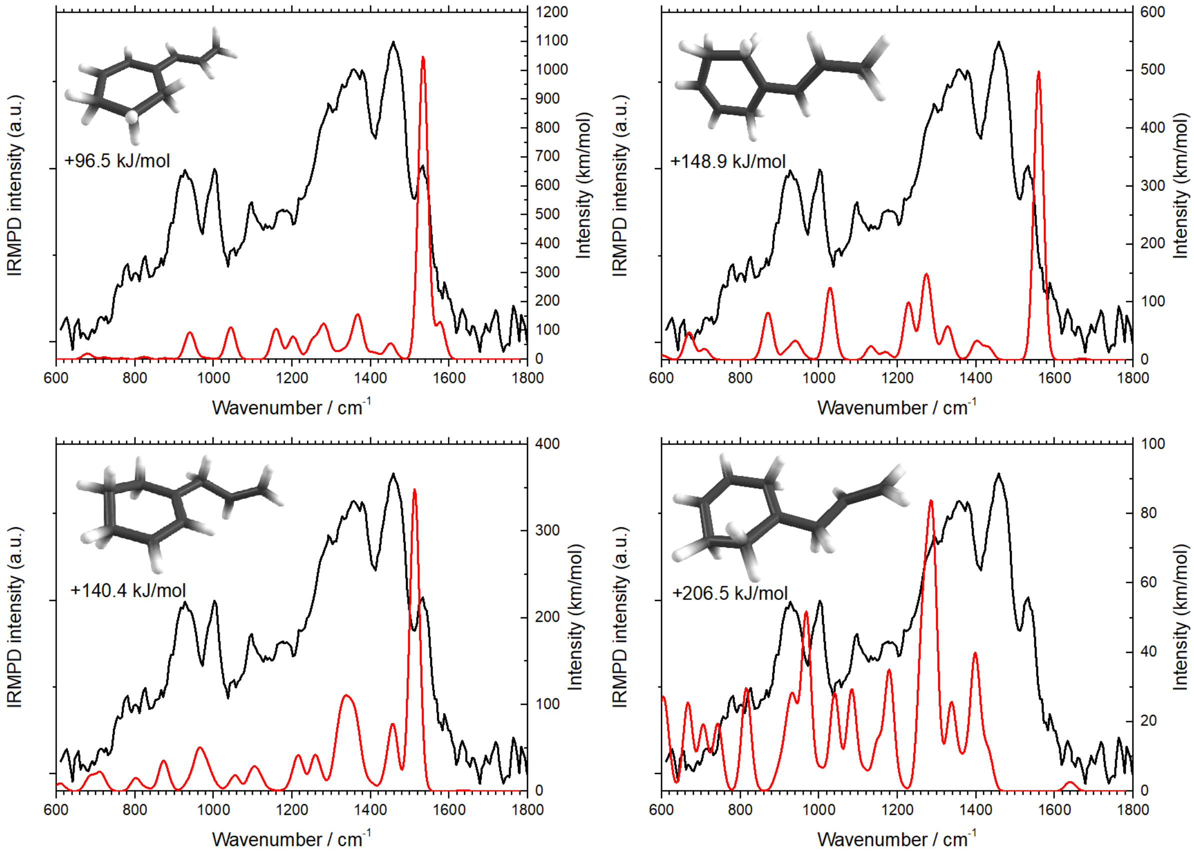

Figure S12: Spectrum of $[M\text{-}CH_3]^+$ formed upon EI of adamantane compared with the DFT computed spectra of various protonated propenylcyclohexadienes.

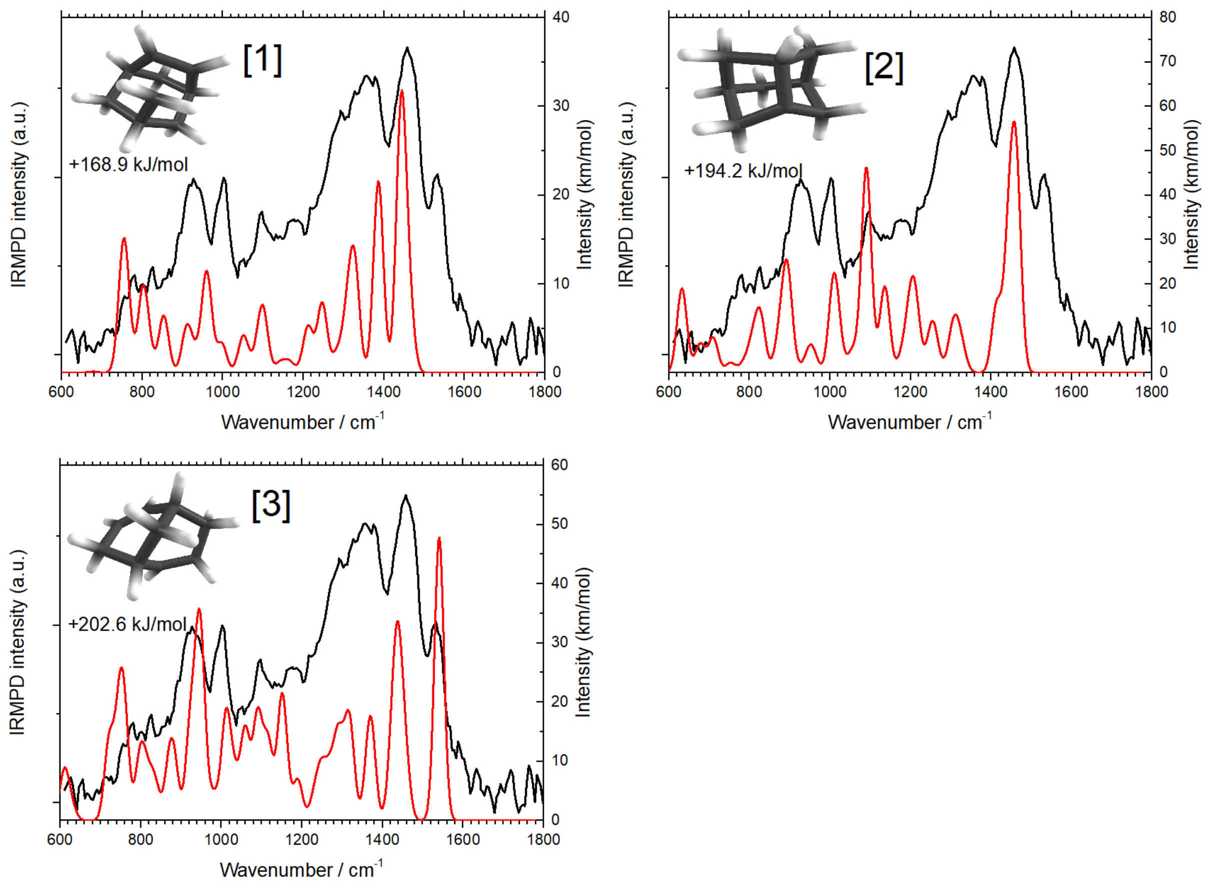

Figure S13: Spectrum of $[M-CH_3]^+$ formed upon EI of adamantane compared with the DFT computed spectra of $C_9H_{13}^+$ cage structures. Detailed optimized structures are available from Table S1-S3.

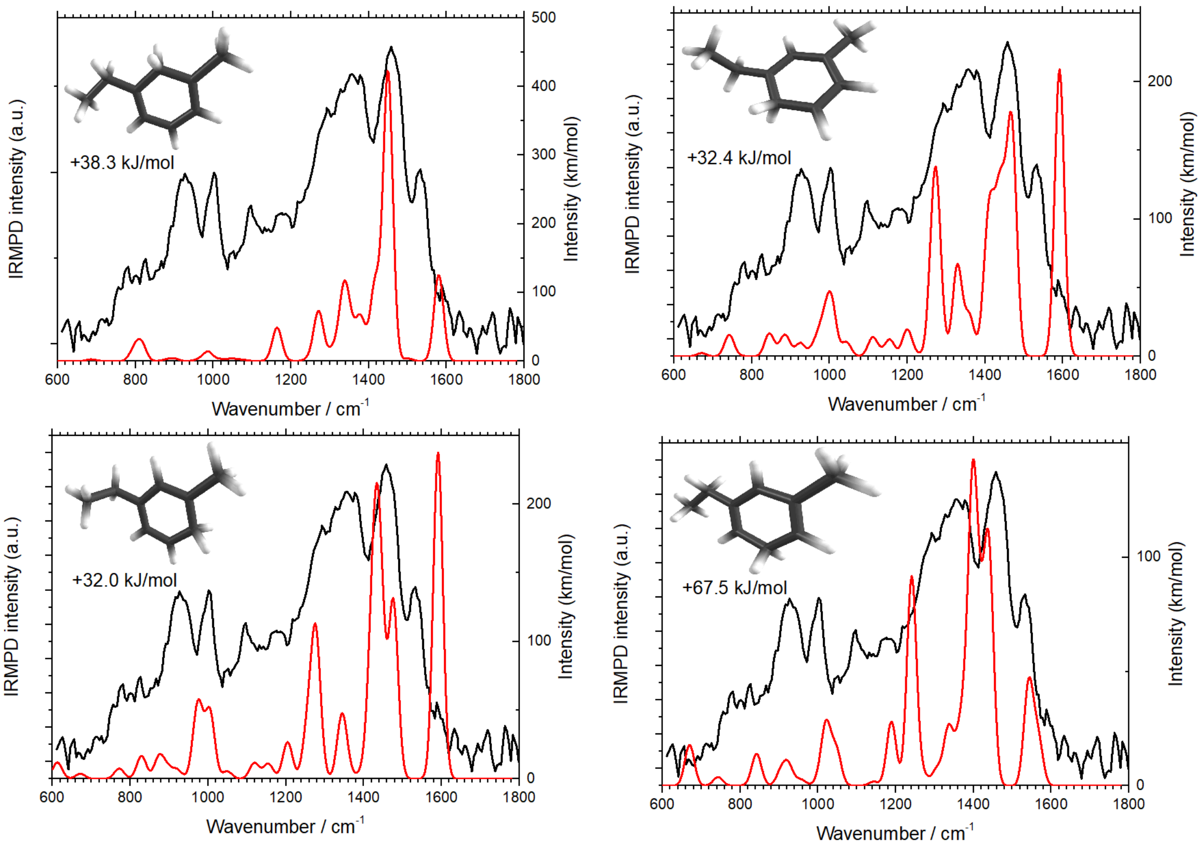

Figure S14: Spectrum of $[M-CH_3]^+$ formed upon electron ionization of adamantane compared with the DFT computed spectra of the various protonated ethylmethylbenzenes.

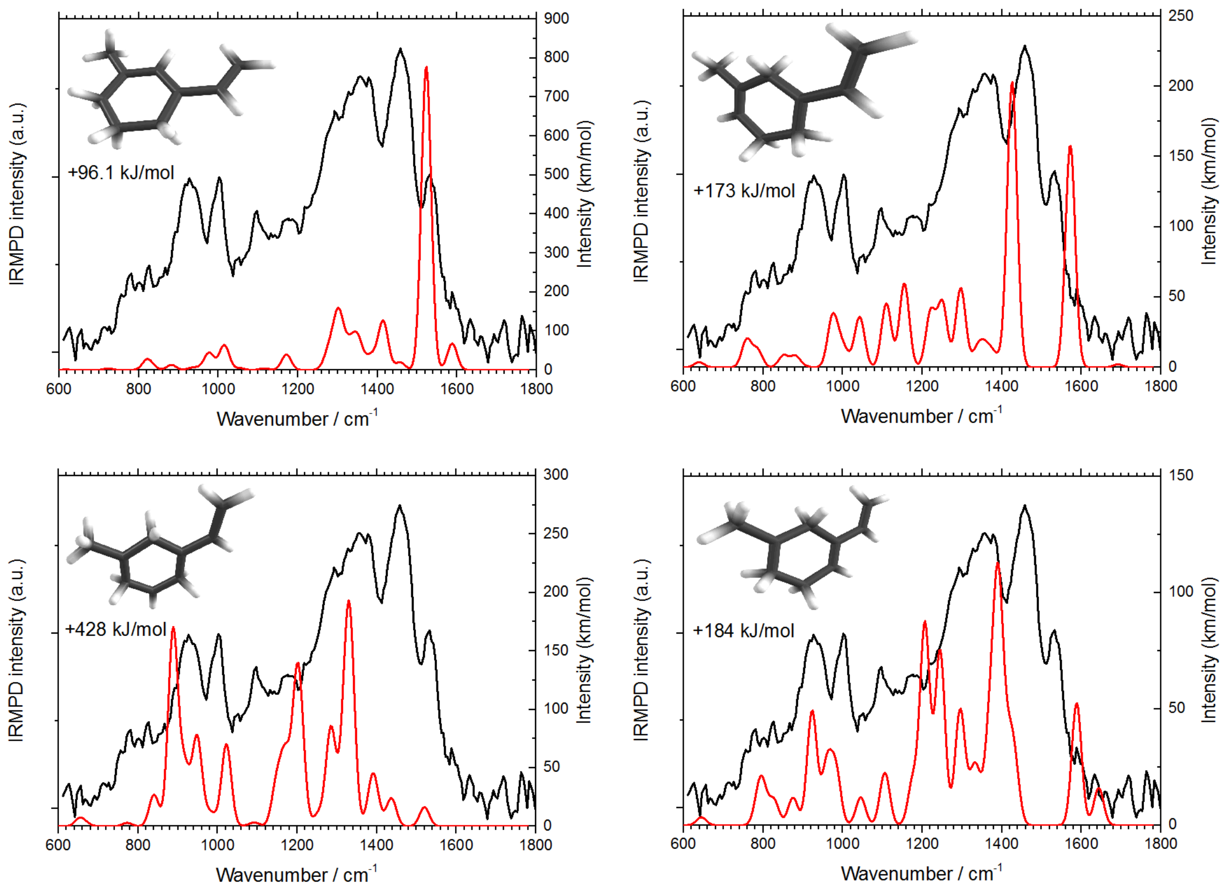

Figure S15: Spectrum of $[M-CH_3]^+$ formed upon electron ionization of adamantane compared with the DFT computed spectra of protonated methylvinylcyclohexadienes.

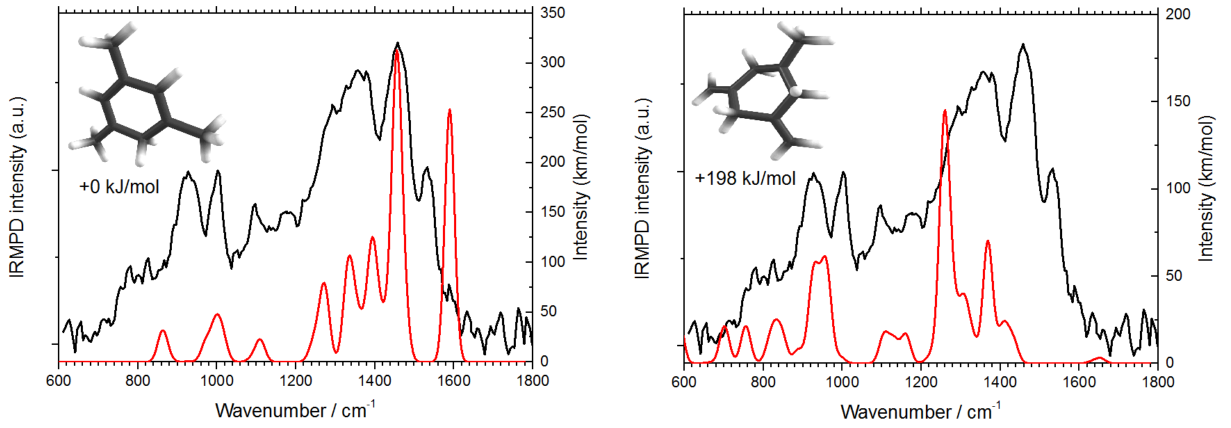

Figure S16: Spectrum of $[M-CH_3]^+$ formed upon electron ionization of adamantane compared with the DFT computed spectra of protonated trimethylbenzene (left) and protonated methyldiisotoluene (right).

Spectrum of the *m/z* 135 fragment

The IRMPD spectrum of the *m/z* 135 fragment is displayed in Figure S14. The best match is found between the measured spectrum and the DFT computed spectrum of 1-adamantyl and the largest mismatch between the two spectra is found in the range around 1000 cm^{-1}. 1-Adamantyl is also the thermodynamically preferred isomer.

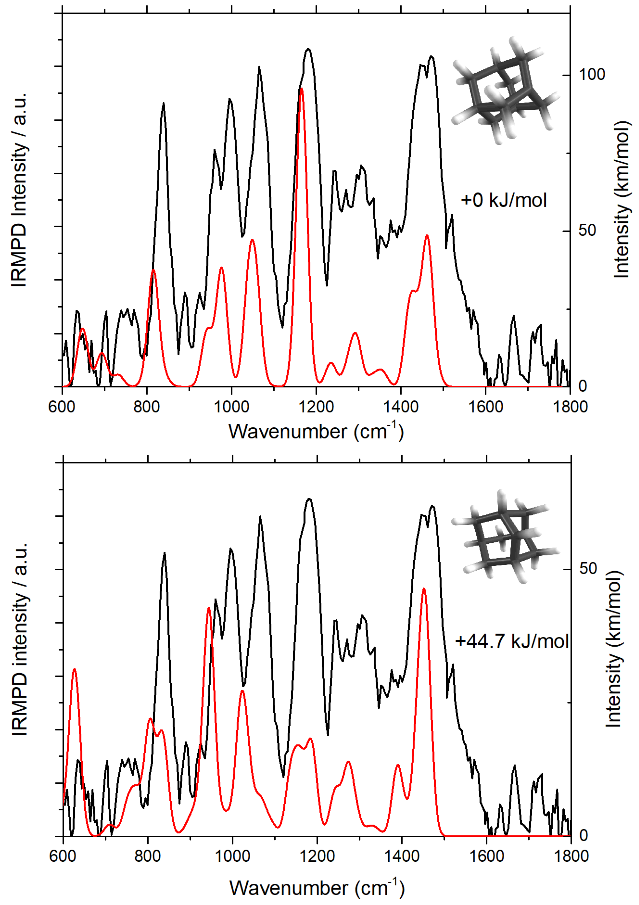

Figure S17: IRMPD spectrum (black) of the m/z=135 ($C_{10}H_{15}^+$) ion formed from the dissociative ionization of adamantane plotted together with the DFT computed spectrum (red) of the 1-adamantyl (top) and 2-adamantyl cation (bottom).

Table S1: Cartesian coordinates for cage structure [1] in Figure S13.

6	1.641444000	0.000013000	-0.561690000
6	0.778913000	1.194658000	-0.083032000
6	0.340519000	0.695329000	1.291999000
6	0.340524000	-0.695353000	1.291985000
6	0.778924000	-1.194650000	-0.083055000
6	-0.505189000	-1.278017000	-0.961493000
6	-1.295072000	0.000001000	-0.620884000
6	-0.505199000	1.278031000	-0.961470000
1	-0.242627000	1.341183000	-2.018443000
1	-1.091571000	2.166553000	-0.712535000
6	-1.389568000	-0.000012000	0.888677000
1	-1.822554000	0.880204000	1.366530000
1	-1.822521000	-0.880250000	1.366519000
1	-2.295960000	0.000000000	-1.060685000
1	-1.091553000	-2.166548000	-0.712575000
1	-0.242617000	-1.341147000	-2.018467000
1	1.304175000	-2.147352000	-0.028670000
1	0.233126000	-1.313246000	2.175287000
1	0.233116000	1.313203000	2.175314000
1	1.304156000	2.147364000	-0.028627000
1	2.617566000	0.000013000	-0.070154000
1	1.805488000	0.000024000	-1.639723000

Table S2: Cartesian coordinates for cage structure [2] in Figure S13.

6	-1.244410000	-1.132660000	-0.622885000
6	-1.276575000	-0.155617000	0.700132000
6	-1.256257000	1.207543000	-0.025817000
6	0.000000000	1.007062000	-0.914781000
6	0.000000000	-0.528287000	-1.026371000
6	1.244412000	-1.132659000	-0.622884000
6	1.276575000	-0.155616000	0.700133000
6	-0.000001000	-0.384654000	1.535997000
1	-0.000001000	-1.396445000	1.951765000
1	-0.000001000	0.301243000	2.389987000
6	1.256256000	1.207543000	-0.025817000
1	1.154642000	2.060744000	0.645002000
1	2.164938000	1.350598000	-0.612857000
1	2.186710000	-0.386265000	1.255125000
1	2.124936000	-0.848924000	-1.199859000
1	1.211783000	-2.200157000	-0.420494000
1	0.000000000	1.547283000	-1.861833000
1	-1.154642000	2.060744000	0.645002000
1	-2.164939000	1.350599000	-0.612857000
1	-2.186712000	-0.386265000	1.255123000
1	-2.124935000	-0.848926000	-1.199860000
1	-1.211782000	-2.200157000	-0.420495000

Table S3: Cartesian coordinates for cage structure [3] in Figure S13.

6	0.073412000	-1.150130000	-0.663152000
6	-0.727456000	-0.970568000	0.588392000
6	-2.014689000	-0.322592000	0.053348000
6	-1.478305000	0.798387000	-0.802559000
6	1.523064000	-0.958086000	-0.702843000
6	1.951404000	0.384885000	-0.023487000
6	0.678149000	1.028602000	0.560869000
6	0.008649000	0.021469000	1.512810000
1	0.756341000	-0.471290000	2.137905000
1	-0.706820000	0.499616000	2.183067000
6	-0.242848000	1.333140000	-0.598097000
1	-2.087534000	1.178521000	-1.617610000
1	0.121607000	2.058219000	-1.321540000
1	0.939324000	1.959545000	1.072124000
1	2.408666000	1.016316000	-0.788746000
1	2.696221000	0.226661000	0.757204000
1	1.928320000	-1.088572000	-1.705353000
1	-2.690496000	0.026069000	0.839610000
1	-2.580559000	-1.025029000	-0.564821000
1	-0.900346000	-1.947398000	1.060306000
1	-0.422289000	-1.622948000	-1.509764000
1	1.909280000	-1.800358000	-0.094064000
